# Antibacterial Activities of Metabolites from *Vitis rotundifolia* (Muscadine) Roots against Fish Pathogenic Bacteria

**DOI:** 10.3390/molecules23112761

**Published:** 2018-10-25

**Authors:** Kevin K. Schrader, Mohamed A. Ibrahim, Howaida I. Abd-Alla, Charles L. Cantrell, David S. Pasco

**Affiliations:** 1 United States Department of Agriculture, Agricultural Research Service, Natural Products Utilization Research Unit, National Center for Natural Products Research, Post Office Box 1848, University, MS 38677, USA; charles.cantrell@ars.usda.gov; 2 Chemistry of Natural Compounds Department, Pharmaceutical and Drug Industries Division, National Research Centre, Dokki, Giza 12622, Egypt; mmibrahi@olemiss.edu (M.A.I.); howaida_nrc@yahoo.com (H.I.A.-A.); 3 National Center for Natural Products Research, School of Pharmacy, University of Mississippi, University, MS 38677, USA; dpasco@olemiss.edu; 4 Department of BioMolecular Sciences, School of Pharmacy, University of Mississippi, University, MS 38677, USA

**Keywords:** antibacterial, channel catfish, columnaris disease, *Flavobacterium columnare*, stilbenes, muscadine, pyranoanthocyanin

## Abstract

Enteric septicemia of catfish, columnaris disease and streptococcosis, caused by *Edwardsiella ictaluri*, *Flavobacterium columnare* and *Streptococcus iniae*, respectively, are the most common bacterial diseases of economic significance to the pond-raised channel catfish *Ictalurus punctatus* industry. Certain management practices are used by catfish farmers to prevent large financial losses from these diseases such as the use of commercial antibiotics. In order to discover environmentally benign alternatives, using a rapid bioassay, we evaluated a crude extract from the roots of muscadine *Vitis rotundifolia* against these fish pathogenic bacteria and determined that the extract was most active against *F. columnare*. Subsequently, several isolated compounds from the root extract were isolated. Among these isolated compounds, (+)-hopeaphenol (**2**) and (+)-vitisin A (**3**) were found to be the most active (bacteriostatic activity only) against *F. columnare*, with 24-h 50% inhibition concentrations of 4.0 ± 0.7 and 7.7 ± 0.6 mg/L, respectively, and minimum inhibitory concentrations of 9.1 ± 0 mg/L for each compound which were approximately 25X less active than the drug control florfenicol. Efficacy testing of **2** and **3** is necessary to further evaluate the potential for these compounds to be used as antibacterial agents for managing columnaris disease.

## 1. Introduction

Two common diseases of channel catfish *Ictalurus punctatus* grown in ponds in the southeastern part of the United States of America (USA) are columnaris disease and enteric septicemia of catfish (ESC) [1,2]. The etiological agent for columnaris disease is the Gram-negative rod-shaped bacterium *Flavobacterium columnare* in the family Flavobacteriaceae [3]. The disease usually results in severe necrosis of gill tissue and skin ulceration from systemic infection. The Gram-negative bacterium *Edwardsiella ictaluri* (Enterobacteriaceae) is the etiological agent for ESC [2]. Gross lesions in channel catfish with ESC can include hemorrhaging at the base of the fins, on the belly, under the jaw and on the backs of infected fish, with small ulcers and/or depigmented lesions. Both diseases have high mortality rates and cost catfish producers millions of U.S. dollars annually [2].

Another common problem in fish species is the bacterial disease Streptococcosis. It can cause heavy economic losses of farmed freshwater fish including hybrid striped bass and tilapias [4]. The Gram-positive bacterium *Streptococcus iniae* is attributed as the cause of streptococcosis, which can result in very high mortality rates in freshwater fish. Catfish producers may manage columnaris disease and ESC by the application of medicated feed containing the antibiotic florfenicol (Aquaflor^®^; Intervet Inc., Millsboro, DE, USA), live attenuated vaccines [5] and nonantibiotic therapeutants such as 35% Perox-Aid^®^ for external columnaris [2]. The potential treatments for columnaris disease with other inorganic agents such as potassium permanganate and copper sulfate pentahydrate have been cited [6]. The disadvantages of these therapeutants are their broad-spectrum toxicity towards non-target organisms (such as channel catfish) [7].

In the USA, only florfenicol (Aquaflor^®^) is approved for the treatment of streptococcal septicemia caused by *S. iniae* in freshwater-reared warm water finfish. Vaccinations may also be a good method for protection against this bacterial infection in Nile tilapia [8].

Because of the limitations of available management approaches for controlling the bacterial species responsible for ESC, columnaris disease and streptococcosis and due to public concerns about environmental impacts from the use of antibiotic-containing feed in agriculture, the discovery of environmentally safe, natural antibacterial compounds would benefit aquaculturists. Previous studies indicated that the *Vitis* species (muscadine) contain large amounts of bioactive phenolics, such as stilbenes, anthocyanins and flavonoids, with some of these compounds possessing antibacterial activities [9,10]. As part of our ongoing efforts to identify such active compounds against isolates of *F. columnare*, *E. ictaluri* and *S. iniae*, we evaluated crude extract and natural compounds from the roots of muscadine (*Vitis rotundifolia* Michx., family Vitaceae) using a rapid bioassay.

## 2. Results and Discussion

Among the three species of fish pathogenic bacteria tested, the crude extract from the roots of *V. rotundifolia* was found to be most active against *F. columnare*, with a 24-h 50% inhibition concentration (IC_50_) of 16.5 ± 6.4 mg/L (clumping of cells can sometimes occur and result in larger variations of results between repeated bioassays) and a minimum inhibitory concentration (MIC) of 10.0 ± 0 mg/L (Table 1). Because the activity was an order of magnitude less active against *S. iniae* compared to *F. columnare* based on the MIC results (100.0 mg/L and 10.0 mg/L, respectively), the bioassay was not repeated for this test bacterial species. The crude extract was not toxic against *E. ictaluri* at the highest test concentration of 100.0 mg/L, therefore, the standard deviation was not calculated. While the relative-to-drug-control-florfenicol (RDCF) values for the 24-h IC_50_ and MIC of the crude extract against *F. columnare* (60.8 mg/L and 100.0 mg/L, respectively) did not indicate strong activity compared to florfenicol, these results are typical for extracts that are compared to isolated pure active compounds because the active compounds are expected to be at lower concentrations in the initial crude extract. Because the crude extract was most active against *F. columnare*, isolated test compounds from the extract were only evaluated against *F. columnare* using the bioassay for the remainder of the study.

**Figure 1:**
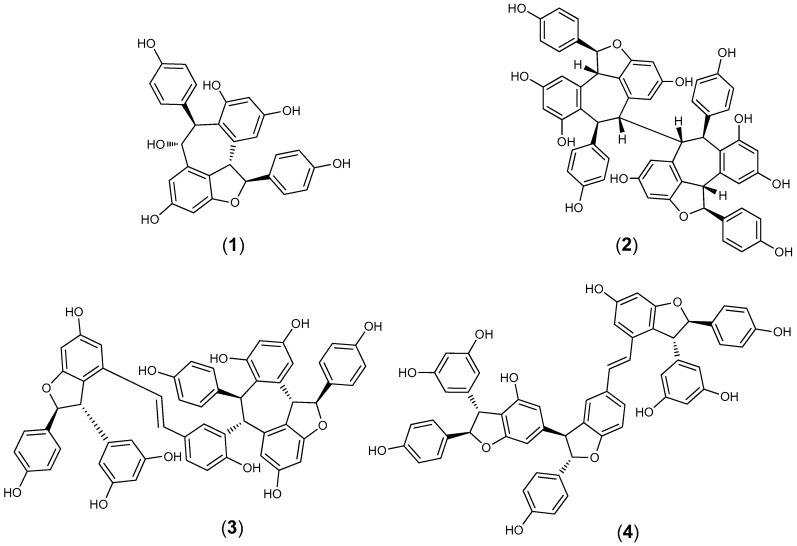
The chemical structures of (+)-ampelopsin (**1**), (+)-hopeaphenol (**2**), (+)-vitisin A (**3**) and (+)-vitisin B (**4**).

Four compounds were isolated from the root crude extract and identified as (+)-ampelopsin A (**1**), (+)-hopeaphenol (**2**), (+)-vitisin A (**3**) and the (+)-enantiomer of vitisin B (**4**) (Figure 1). Based on 24-h IC_50_ results, compounds **2** and **3** were the most active against *F. columnare*, with 24-h IC_50_ of 4.0 ± 0.7 and 7.7 ± 0.6 mg/L, respectively (Table 2). Based on the 24-h IC_50_ results, compound **2** was found to be slightly more active against the pathogenic bacterium *F. columnare* than **3**. Subsequently, the 24-h IC_50_ RDCF value of 6.8 for **2** also indicated strong activity and the 24-h IC_50_ MTT of 8.9 ± 0.3 mg/L for **2** indicated less viable cells remaining compared to **3** (24-h IC_50_ MTT = 16.3 ± 0 mg/L). Compound **1**, stilbene oligomer (viniferin), was not active against *F. columnare* at the highest test concentration of 47.0 mg/L. Compound **4** was less active than **2** and **3** against *F. columnare* based on 24-h IC_50_ results and the MTT bioassay indicated no reduction in viable cells even at the highest test concentration of 90.7 mg/L.

The minimum bactericidal concentration (MBC) results for each test compound indicated no bactericidal activity against *F. columnare* at even the highest test concentrations (i.e., MBC > 100 µM). Therefore, the activity of compound **2** can be considered as bacteriostatic rather than bactericidal at the concentrations evaluated. 

Stilbenes from the Vitaceae are thought to play a role in both animal and human health including their antimicrobial activity. Therefore, these compounds have been the subject of numerous studies during the past decade. They were reported to have activities against various pathogens, such as *Plasmopara viticola*, *Cladosporium cuccumerinum*, *Plasmopara viticola* and *Sphaeropsis sapinea* [11]. The hopeaphenol class of polyphenols are tetramers of resveratrol which is a *trans*-stilbene demonstrated to possess antibacterial activity against certain human pathogenic bacteria [12]. A previous study evaluated the antibacterial activity of (−)-hopeaphenol against ten animal and plant pathogenic bacteria (e.g., Gram-negative *Yersinia pseudotuberculosis* and *Pseudomonas aeruginosa*) but found no significant growth inhibition at test concentrations as high as 90.7 mg/L (100 µM) [13]. However, our current study demonstrated growth inhibition of *F. columnare* by (+)-hopeaphenol (**2**) at 9.1 mg/L (Table 2). The researchers in the previous study [13] suggested that low cell permeability due to the size and molecular weight of (−)-hopeaphenol and its subsequent interaction with bacterial secretion systems (e.g., toxin delivery system T3SS) at the cell surface rather than growth inhibition as the approach for targeting bacterial virulence. Our results with *F. columnare* indicate growth inhibition can occur at lower concentrations of (+)-hopeaphenol (**2**).

The pyranoanthocyanin vitisin A (**3**) has previously been isolated and identified from extracts of the grapevines *Vitis coignetiae* Pulliat. and *Vitis vinifera* L. (Vitaceae) [14] and from extracts of the roots of the grapevine *Vitis thunbergii* Siebold & Zucc. (Vitaceae) [15]. Although the antiplatelet and antioxidative activities of vitisin analogs were reported, specific antibacterial activities were not studied [15]. 

Efficacy studies of (+)-hopeaphenol (**2**) and (+)-vitisin A (**3**) as additives to fish feed and/or as therapeutants still needs to be performed to further evaluate their potential use in managing columnaris disease. Vitisin A (**3**) has been cited as a strong hepatoxic constituent of *Vitis coignetiae* [14], therefore, careful examination of the potential adverse health effects of vitisin A (**3**) on fish prior to any potential efficacy studies as an antibacterial compound against columnaris disease would need to be performed.

## 3. Materials and Methods

### 3.1. Plant Material

The crude root extract of *Vitis rotundifolia* in 95% EtOH (NPID 127513, 50 mg) was provided through the repository of the National Center for Natural Products Research, School of Pharmacy, University of Mississippi, University, MS, USA. The original specimen was collected in 2007 from a forest near Leon, FL, USA.

### 3.2. Extraction and Isolation

Samples of root from *V. rotundifolia* were placed in cells of an accelerated extraction system (ASE 300; Thermo Fisher Scientific, Waltham, MA, USA) and extracted with 95% EtOH three times and for 10 min per extraction. Approximately 250 mg of the 95% EtOH root extract was dissolved in 2000 μL of methanol and then exposed to HPLC separation which was conducted using Waters Prep 4000 HPLC system equipped with a UV-Diode detector (2996, Agilent Technologies, Inc., Santa Clara, CA, USA) controlled by Empower software (Rev. A. 10.02, Agilent Technologies, Inc., Santa Clara, CA, USA). The analysis of the extract was carried out on a RP-C18 column (250 × 21.2 mm; particle size 10 µm; Luna) at 25 °C and using the gradient system of eluent water, 0.1% AcOH (A) and acetonitrile, 0.1% AcOH (B) for the separation of target compounds. The gradient condition was as follows: 0–2 min (10% B), 2–45 min (10% B to 60% B) and 45–50 min (60% B to 100% B). The flow rate of the solvent was 10.0 mL/min and the injection volumes were 400 µL. All separations were carried out at wavelengths of 254, 280 and 325 nm with a run time of 50 min. Compounds **1**–**4** eluted at 28, 35, 38 and 42 min, respectively. NMR spectra were acquired on a Bruker 400 MHz NMR spectrometer (Bruker, Billerica, MA, USA) at 400 (^1^H) and 100 MHz (^13^C) in CD_3_OD using the residual solvent as an internal standard (Supporting Information). Multiplicity determinations (DEPT) and 2D NMR spectra (HMQC, HMBC, NOESY) were obtained using standard Bruker pulse programs (Bruker III HD, Billerica, MA, USA). Acquisition of high-resolution mass data was acquired using AccuTOF (JMS-T100LC). Comparing the NMR data of the isolated metabolites with the previously reported has confirmed their identities as (+)-ampelopsin A (**1**), (+)-hopeaphenol (**2**) and (+)-vitisin A (**3**) [14,16,17]. The NMR data of compound (**4**) matched with the reported data for vitisin B [18], however it showed positive optical rotation indicating its identity as the (+)-enantiomer of vitisin B (**4**).

*(+)-Ampelopsin A* (**1**). For ^1^H and ^13^C-NMR data, see Supporting Information [17]. High-resolution ESI/MS: *m/z* 493.13312 [M + Na]^+^; calculated for C_28_H_22_NaO_7_, 493.12185. [*α*]^25^_D_ + 183 (*c* 0.1, MeOH).

*(+)-Hopeaphenol* (**2**). For ^1^H and ^13^C-NMR data, see Supporting Information [16]. High-resolution ESI/MS: *m/z* 929.26922 [M + Na]^+^; calculated for C_56_H_42_NaO_12_, 929.25293. [*α*]^25^_D_ + 201 (*c* 0.1, MeOH).

*(+)-Vitisin A* (**3**). For ^1^H and ^13^C-NMR data, see Supporting Information [14]. High-resolution ESI/MS: *m/z* 930.26681 [M + Na + H]^2+^; calculated for C_56_H_43_NaO_12_, 930.26522. [*α*]^25^_D_ + 204 (*c* 0.1, MeOH).

*(+)-Vitisin B* (**4**). For ^1^H and ^13^C-NMR data, see Supporting Information [18]. High-resolution ESI/MS: *m/z* 930.26734 [M + Na + H] ^2+^; calculated for C_56_H_43_NaO_12_, 930.26522. [*α*]^25^_D_ + 55 (*c* 0.1, MeOH).

### 3.3. Microorganisms and Culture Material

The bacterial isolate of *F. columnare* [isolate ALM-00-173 (genomovar II)] was obtained from Dr. Covadonga Arias (Department of Fisheries and Allied Aquacultures, Auburn University, Auburn, AL, USA). In order to assure purity, cultures of *F. columnare* ALM-00-173 were maintained separately on modified Shieh (MS) agar plates (pH 7.2–7.4) at 29 ± 1 °C [19]. Prior to conducting the bioassay, individual colonies of *F. columnare* ALM-00-173 were used to prepare assay culture material by culturing in 75 mL of MS broth for at least 24 h at 29 ± 1 °C at 150 rpm on a rotary shaker (model C24KC; New Brunswick Scientific, Edison, NJ, USA). After overnight incubation, a 0.5 McFarland standard of *F. columnare* ALM-00-173 culture material was made by micropipetting cells from the broth culture to fresh MS broth [20]. 

The isolate of *E. ictaluri* (isolate S02-1039) was obtained from Mr. Tim Santucci (formerly with the College of Veterinary Medicine, Mississippi State University, Stoneville, MS, USA), and cultures of *E. ictaluri* were maintained at 29 ± 1 °C on 3.8% Mueller-Hinton (MH) agar plates (pH 7.3) (Becton, Dickinson and Company, Sparks, MD, USA) in order to assure purity. Prior to performing the bioassay, single colonies of *E. ictaluri* S02-1039 were used to prepare assay culture material by aseptically transferring bacterial cells from colonies on agar plates to 45 mL of 3.8% MH broth in order to produce a bacterial cell density of 0.5 McFarland standard.

A culture of *S. iniae* (isolate LA94-426) was provided by Dr. Ahmed Darwish (formerly with the U.S. Department of Agriculture, Agricultural Research Service, Harry K. Dupree Stuttgart National Aquaculture Research Center, Stuttgart, AR, USA). In order to assure purity, cultures of *S. iniae* LA94-426 were maintained at 29 ± 1 °C on agar plates of Columbia CNA containing 5% sheep blood (Remel, Inc., Lenexa, KS, USA). The bioassay culture material of *S. iniae* LA94-426 was prepared in the same manner used for *F. columnare* ALM-00-173, except 3.8% MH broth was utilized and broth cultures were incubated for 18 h prior to preparing the 0.5 MacFarland standard.

### 3.4. Antibacterial Bioassay

The crude extract from the roots of *V. rotundifolia* and isolated test compounds were evaluated for antibacterial activity using a rapid 96-well microplate bioassay [20]. Florfenicol was utilized as a positive drug control and control wells were included in which no test material was added. The crude extract and test compounds were dissolved separately in technical grade 100% methanol while florfenicol was dissolved in technical grade 100% ethanol. The final test concentrations of the crude extract were 0.001, 0.01, 0.1, 1.0, 10.0, and 100.0 mg/L. Final concentrations of test compounds and florfenicol were 0.01, 0.1, 1.0, 10.0 and 100.0 µM. Three replications were used for each dilution of the crude extract, each test compound and florfenicol. Final results were converted to units of mg/L to allow comparisons with previous studies.

The 24-h 50% inhibition concentration (IC_50_) and minimum inhibitory concentration (MIC) were determined using sterile 96-well polystyrene microplates (Corning Costar Corp., Acton, MA, USA) with flat-bottom wells. Crude extract, dissolved test compounds and florfenicol were initially micropippeted into separate microplate wells (10 µL/well), and the solvent was completely evaporated before 0.5 MacFarland bacterial culture was added to the microplate wells (200 µL/well). Microplates were incubated at 29 ± 1 °C (VWR model 2005 incubator; Sheldon Manufacturing Inc., Cornelius, OR, USA). A Packard model SpectraCount microplate photometer (Packard Instrument Company, Meriden, CT, USA) was used to measure the absorbance (630 nm) of the microplate wells at time 0 and after 24 h of incubation.

After 24 h of incubation, the cell viability of *F. columnare* was determined for the test compounds by using 3(4,5-dimethylthiazol-2-yl)-2,5-diphenyl tetrazolium bromide (MTT) (GenScript, Piscataway, NJ, USA) and previous procedures [20]. For the MTT bioassay, 40 µL of culture material from each growth-assay microplate well were aseptically micropipetted to a corresponding well in another sterile 96-well polystyrene microplate containing 10 µL of MTT (50 mg/10 mL phosphate buffered saline) per well. Each microplate was maintained for 4 h at 29 ± 1 °C and then 50 µL of lysing buffer [20% sodium dodecyl sulfate in 50% *N*,*N*-dimethylformamide (pH 4.7)] was added to each well. Microplates were then incubated for 20 h after which absorbance (570 nm) was measured (without mixing) using a Packard model SpectraCount microplate photometer. Microplate wells containing 3.8% MH broth, MTT and lysing buffer were used as blanks.

Means and standard deviations of absorbance measurements were calculated, graphed and compared to controls to determine the 24-h IC_50_ and MIC for the crude extract and each test compound [20]. The 24-h IC_50_ and MIC results for the crude extract and each test compound were divided by the respective 24-h IC_50_ and MIC results obtained for the drug control florfenicol to determine the relative-to-drug-control florfenicol (RDCF) values.

The minimum bactericidal concentration (MBC) of isolated compounds was determined as outlined previously [20]. Briefly, 5 µL of culture material were aseptically transferred from each growth bioassay microplate well to 3.8% MH agar plates and these plates were incubated at 29 ± 1 °C for 24 h. Plates were visually evaluated for growth and the MBC was determined to be the lowest concentration in which no growth was present on the agar plate. 

## 4. Conclusions

We isolated four compounds from the root extract of *V*. *rotundifolia*. Among these compounds, (+)-hopeaphenol and (+)-vitisin A were found to possess the strongest activity against the fish pathogenic bacterium *F*. *columnare*. Although these two compounds possess bacteriostatic activity only, efficacy testing will determine the potential of these two compounds for use in the management of columnaris disease.

## Figures and Tables

**Table 1:** Results of the bioassay evaluation of the crude extract from the roots of *Vitis rotundifolia* against fish pathogenic bacteria.

Bacteria Species	24-h IC_50_^a^ (mg/L)	MIC ^b^ (mg/L)	24-h IC_50_ RDCF ^c^	MIC RDCF ^c^
*F. columnare*	16.5 (6.4)	10.0 (0)	60.8 (32.9)	100.0 (0)
*E. ictaluri*	>100.0	>100.0	ND ^d^	ND ^d^
*S. iniae*	22.0 (0)	100.0 (0)	220.0 (0)	>1000.0

^a^ 24-h IC_50_ = 50% inhibition concentration, ^b^ MIC = Minimum inhibitory concentration, ^c^ RDCF = Relative-to-drug-control florfenicol; values below 1.0 indicate higher antibacterial activity compared to florfenicol. Mean 24-h IC_50_ and MIC values ± standard deviation (SD) for florfenicol were 24-h IC_50_ = 0.4 ± 0.1 mg/L and MIC = 0.1 ± 0 mg/L. ^d^ ND = not determined. Numbers in parentheses are the SD of the mean.

**Table 2:** The bioassay evaluation of compounds isolated from the crude extract of the roots of *V. rotundifolia* against *F. columnare*.

Test Compound	24-h IC_50_^a^	MIC ^b^	24-h IC_50_ RDCF ^c^	MIC RDCF ^c^	24-h IC_50_ MTT ^d^
Florfenicol	0.6 (0)	0.4 (0)			
**1**	>47.0	>47.0	ND ^e^	ND ^e^	ND ^e^
**2**	4.0 (0.7)	9.1 (0)	6.8 (0)	25.3 (0)	8.9 (0.3)
**3**	7.7 (0.6)	9.1 (0)	13.1 (0)	25.3 (0)	16.3 (0)
**4**	41.3 (5.8)	9.1 (0)	70.0 (0)	25.3 (0)	>90.7

^a^ 24-h IC_50_ = 50% inhibition concentration in mg/L, ^b^ MIC = Minimum inhibitory concentration in mg/L, ^c^ RDCF = Relative-to-drug-control florfenicol; values below 1.0 indicate higher antibacterial activity compared to florfenicol, ^d^ MTT (cell viability) portion of the bioassay, ^e^ ND = not determined. Numbers in parentheses are the standard deviation of the mean.

## Supplementary Materials

Supporting information is available online.

## References

[1] Plumb J.A., Hanson L.A. (2011). Health Maintenance and Principal Microbial Diseases of Cultured Fishes.

[2] Wagner B.A. (2002). The epidemiology of bacterial diseases in food-size channel catfish. J. Aquat. Anim. Health.

[3] Durborow R.M., Thune R.L., Hawke J.P., Camus A.C. (1998). Columnaris Disease: A Bacterial Infection Caused by Flavobacterium columnare.

[4] Shoemaker C.A., Klesius P.H., Evans J.J. (2001). Prevalence of *Streptococcus iniae* in tilapia, hybrid striped bass, and channel catfish on commercial fish farms in the United States. Am. J. Vet. Res..

[5] Klesius P., Evans J., Shoemaker C. (2006). A US perspective on advancements in fish vaccine development. Aquac. Health Int..

[6] Plumb J.A. (1999). Health Maintenance and Principal Microbial Diseases of Cultured Fishes.

[7] Boyd C.E., Tucker C.S. (1998). Pond Aquaculture Water Quality Management.

[8] Shoemaker C.A., LaFrentz B.R., Klesius P.H., Evans J.J. (2010). Protection against heterologous *Streptococcus iniae* isolates using a modified bacterin vaccine in Nile tilapia, *Oreochromis niloticus* (L.). J. Fish. Dis..

[9] Peng S.C., Cheng C.Y., Sheu F., Su C.H. (2008). The antimicrobial activity of heyneanol A extracted from the root of taiwanese wild grape. J. Appl. Microbiol..

[10] Park Y.J., Biswas R., Phillips R.D., Chen J. (2011). Antibacterial activities of blueberry and muscadine phenolic extracts. J. Food Sci..

[11] Jeandet P., Douillet-Breuil A.C., Bessis R., Debord S., Sbaghi M., Adrian M. (2002). Phytoalexins from the Vitaceae: Biosynthesis, phytoalexin gene expression in transgenic plants, antifungal activity, and metabolism. J. Agric. Food. Chem..

[12] Paulo L., Ferreira S., Gallardo E., Queiroz J.A., Domingues F. (2010). Antimicrobial activity and effects of resveratrol on human pathogenic bacteria. World J. Microbiol. Biotechnol..

[13] Zetterström C.E., Hasselgren J., Salin O., Davis R.A., Quinn R.J., Sundin C., Elofsson M. (2013). The resveratrol tetramer (-)-hopeaphenol inhibits type III secretion in the Gram-negative pathogens *Yersinia pseudotuberculosis* and *Pseudomonas aeruginosa*. PLoS ONE.

[14] Ito J., Gobaru K., Shimamura T., Niwa M., Takaya Y., Oshima Y. (1998). Absolute configurations of some oligostilbenes from *Vitis coignetiae* and *Vitis vinifera* ‘Kyohou’. Tetrahedron.

[15] Huang Y.-L., Tsai W.-J., Shen C.-C., Chen C.-C. (2005). Resveratrol derivatives from the roots of *Vitis thunbergii*. J. Nat. Prod..

[16] Ito J., Niwa M., Oshima Y. (1997). A new hydroxystilbene tetramer named isohopeaphenol from *Vitis vinifera* ’Kyohou’. Heterocycles.

[17] Chen L.G., Wang C.C. (2009). Preparative separation of oligostilbenes from *Vitis thunbergii* var. *taiwaniana* by centrifugal partition chromatography followed by Sephadex LH-20 column chromatography. Sep. Purif. Technol..

[18] Oshima Y., Kamijou A., Ohizumi Y., Niwa M., Ito J., Hisamichi K., Takeshita M. (1995). Novel oligostilbenes from *Vitis coignetiae*. Tetrahedron.

[19] Decostere A., Henckaerts K., Haesebrouck F. (2002). An alternative model to study the association of rainbow trout (*Oncorhynchus mykiss* L.) pathogens with the gill tissue. Lab. Anim..

[20] Schrader K.K., Harries M.D. (2006). A rapid bioassay for bactericides against the catfish pathogens *Edwardsiella ictaluri* and *Flavobacterium columnare*. Aquac. Res..

