# Supplementary material for: Antibacterial Activities of Metabolites from Vitis rotundifolia (Muscadine) Roots against Fish Pathogenic Bacteria
Source: Molecules. 2018 Oct 25;23(11):2761. doi: 10.3390/molecules23112761 (PMC6278413; doi:10.3390/molecules23112761)
Supplement: Supplementary file 1 [file molecules-23-02761-s001.zip › molecules-366901-SI.pdf]

Kevin K. Schrader[a*], Mohamed A. Ibrahim[b,c], Howaida I. Abd-Alla[b], Charles L. Cantrell[a], and David S. Pasco[c]

[a]United States Department of Agriculture, Agricultural Research Service, Natural Products Utilization Research Unit, National Center for Natural Products Research, Post Office Box 1848, University, Mississippi, USA

[b]Chemistry of Natural Compounds Department, Pharmaceutical and Drug Industries Division, National Research Centre, Post Office Box 12622, Dokki, Giza, Egypt

[c]National Center for Natural Products Research, School of Pharmacy, University of Mississippi, University, Mississippi, USA

*To whom correspondence should be addressed. Tel: +1 662-915-1144. Fax: +1 662-915-1035. E-mail: kevin.schrader@ars.usda.gov

Table of contents:

Fig.	Title	Page
SI 1	^{1}H NMR spectrum of (+)-Ampelopsin A [CD_3OD]	4
SI 2	^{13}C NMR spectrum of (+)-Ampelopsin A [CD_3OD]	5
SI 3	^{1}H NMR spectrum of (+)-Hopeaphenol [CD_3OD]	6
SI 4	^{13}C NMR spectrum of (+)-Hopeaphenol [CD_3OD]	7
SI 5	^{1}H NMR spectrum of (+)-Vitisin A [CD_3OD]	8
SI 6	^{13}C NMR spectrum of (+)-Vitisin A [CD_3OD]	9
SI 7	^{1}H NMR spectrum of (+)-Vitisin B [CD_3OD]	10
SI 8	^{13}C NMR spectrum of (+)-Vitisin B [CD_3OD]	11

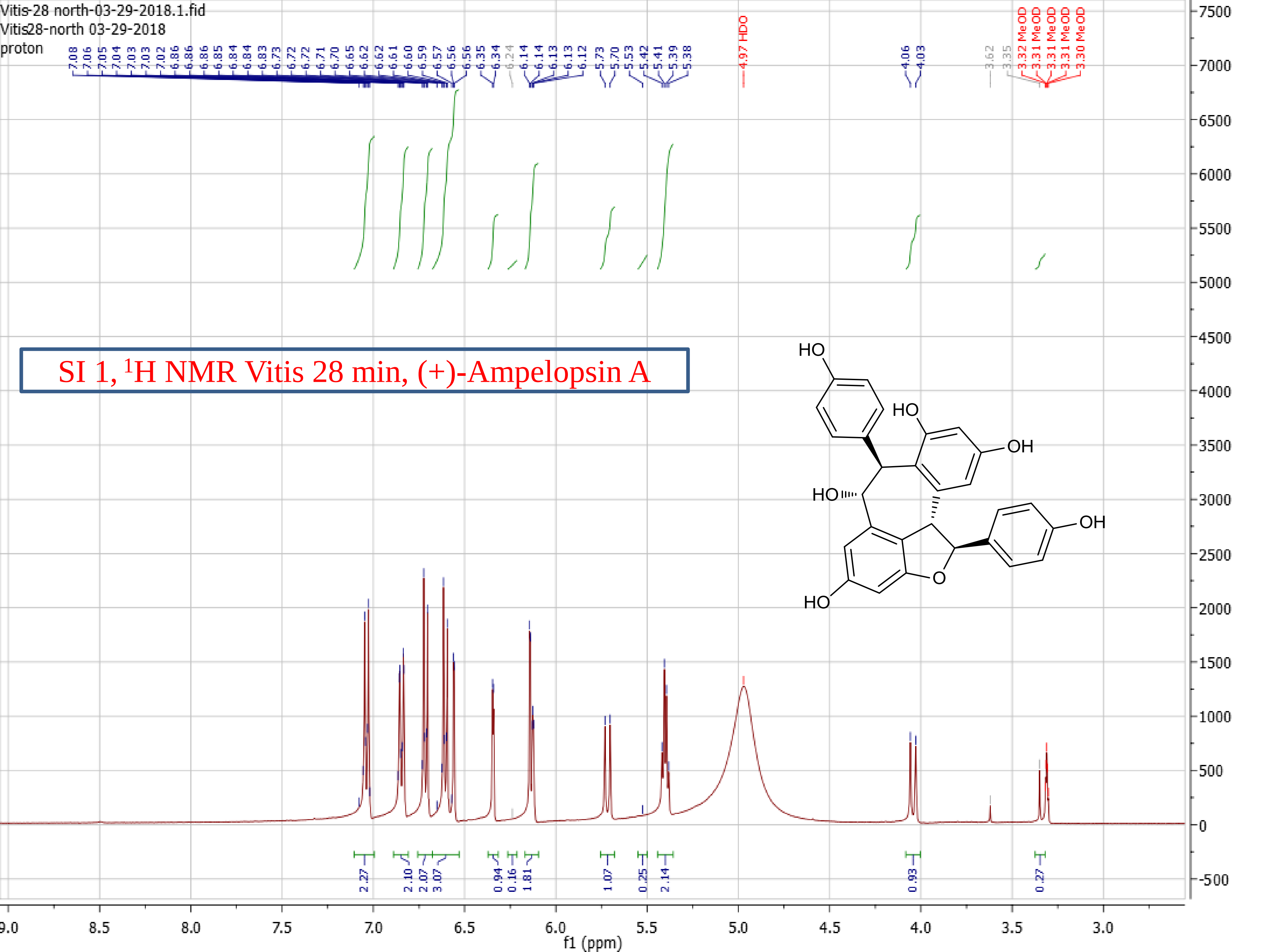

SI 1, ^{1}H NMR Vitis 28 min, (+)-Ampelopsin A

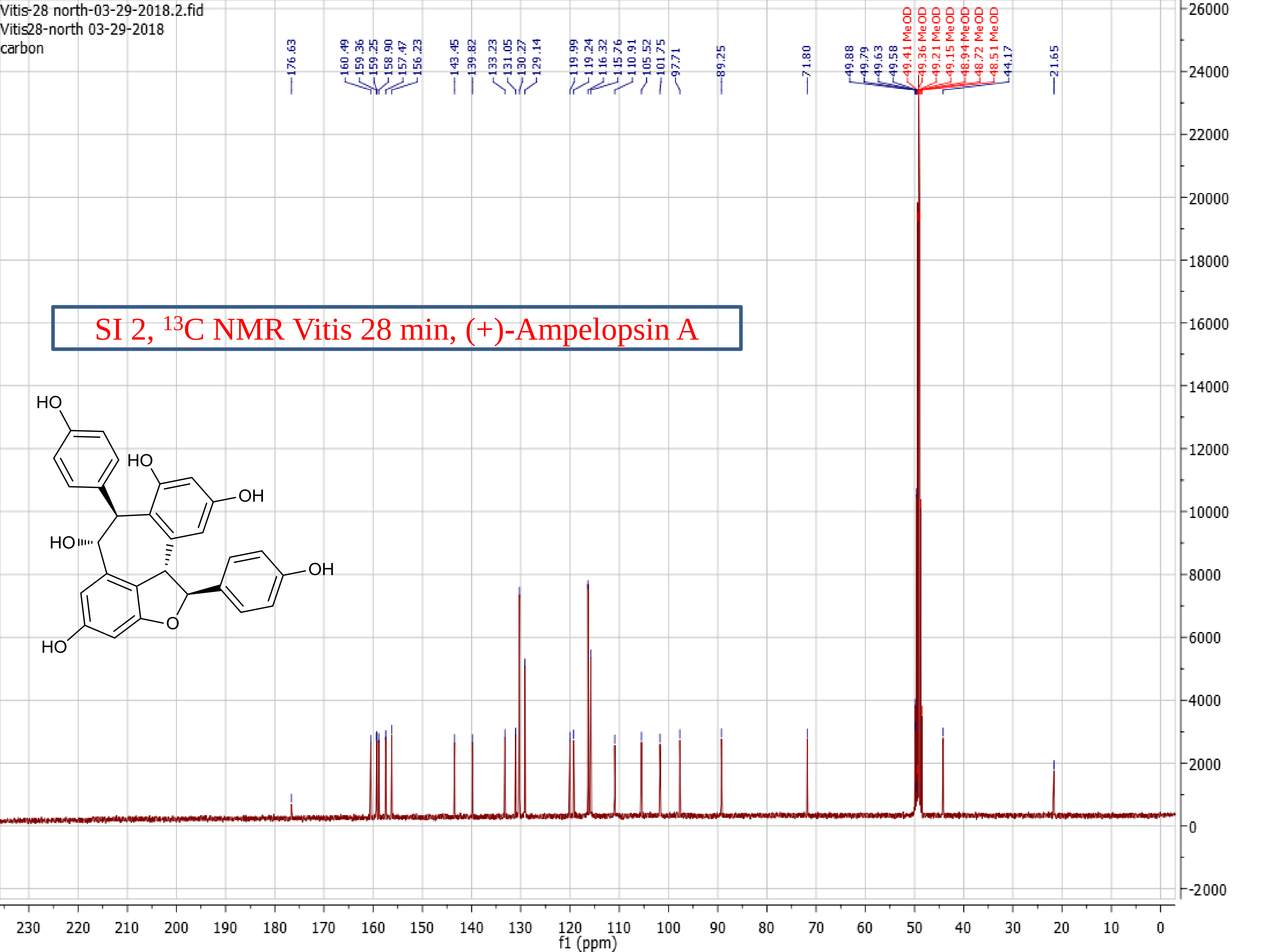

SI 2, ^{13}C NMR Vitis 28 min, (+)-Ampelopsin A

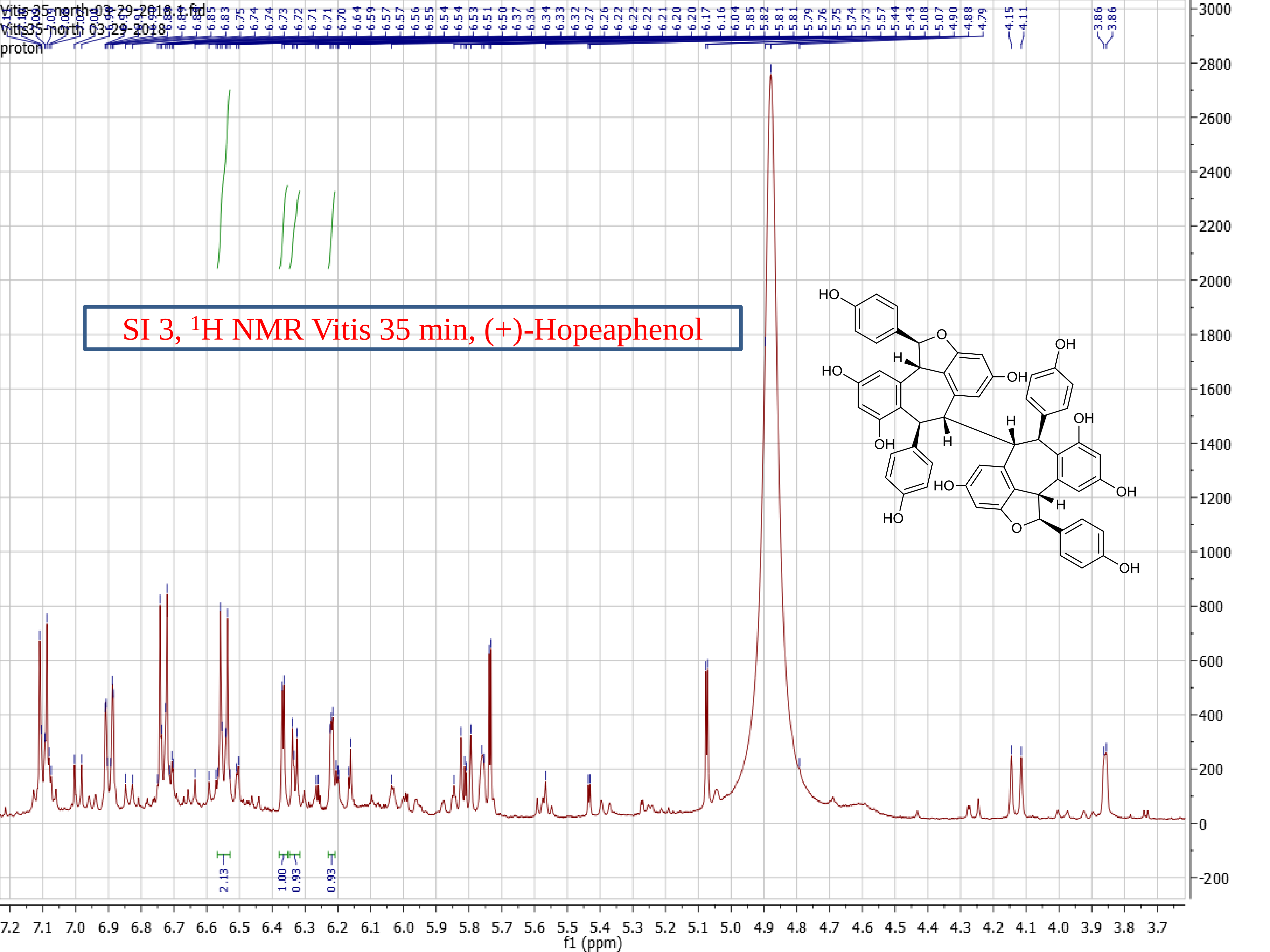

SI 3, ^{1}H NMR Vitis 35 min, (+)-Hopeaphenol

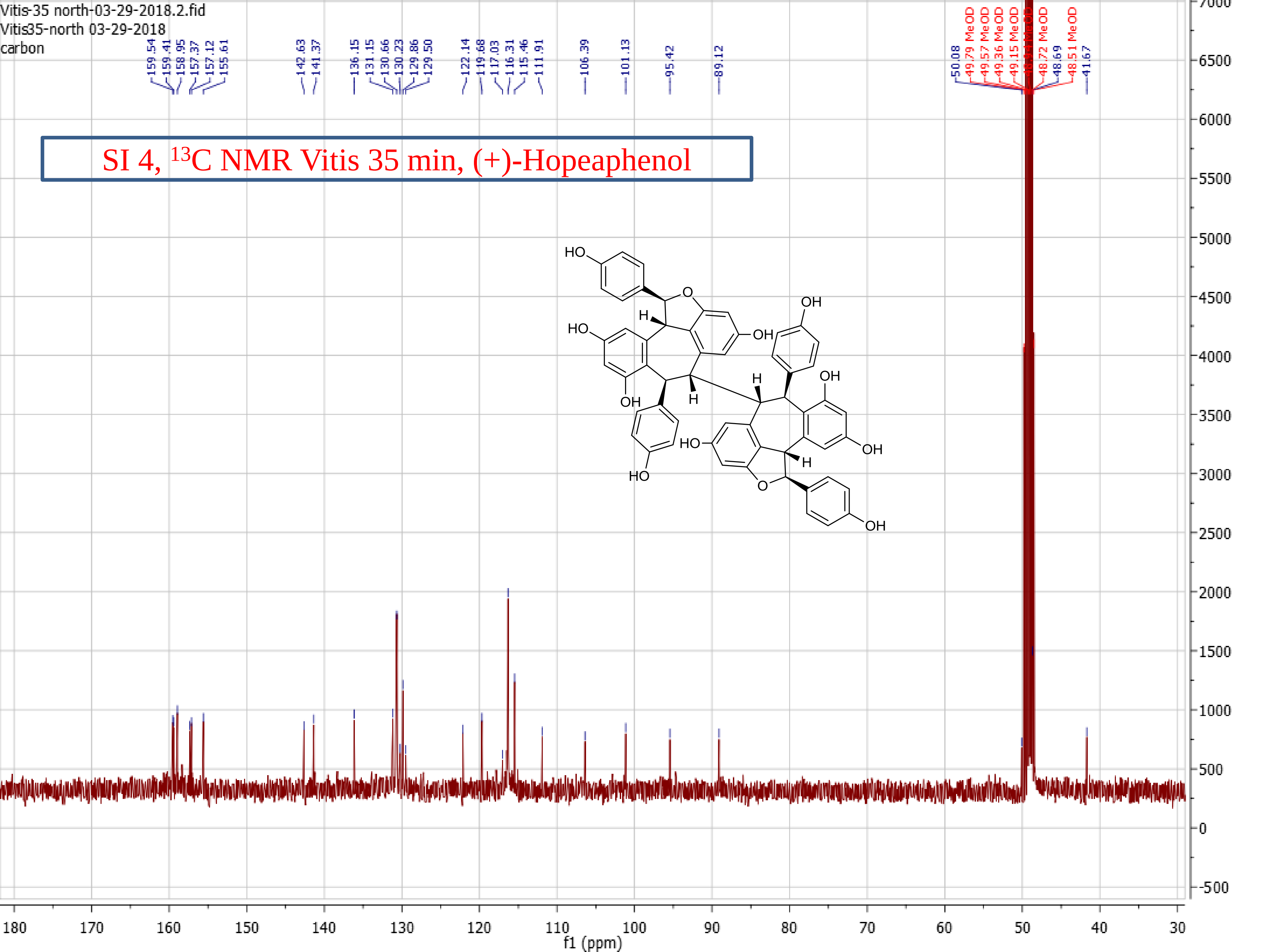
Vitis-35 north-03-29-2018.2.fid
Vitis35-north 03-29-2018
carbon
159.54
159.41
158.95
157.37
157.12
155.61
142.63
141.37
136.15
131.15
130.66
130.23
129.86
129.50
122.14
119.68
117.03
116.31
115.46
111.91
106.39
101.13
95.42
89.12
50.08
49.79 MeOD
49.57 MeOD
49.36 MeOD
49.15 MeOD
48.72 MeOD
48.69
48.51 MeOD
41.67
SI 4, 13C NMR Vitis 35 min, (+)-Hopeaphenol
HO
O
H
OH
HO
OH
OH
H
H
OH
HO
HO
OH
H
O
OH
f1 (ppm)

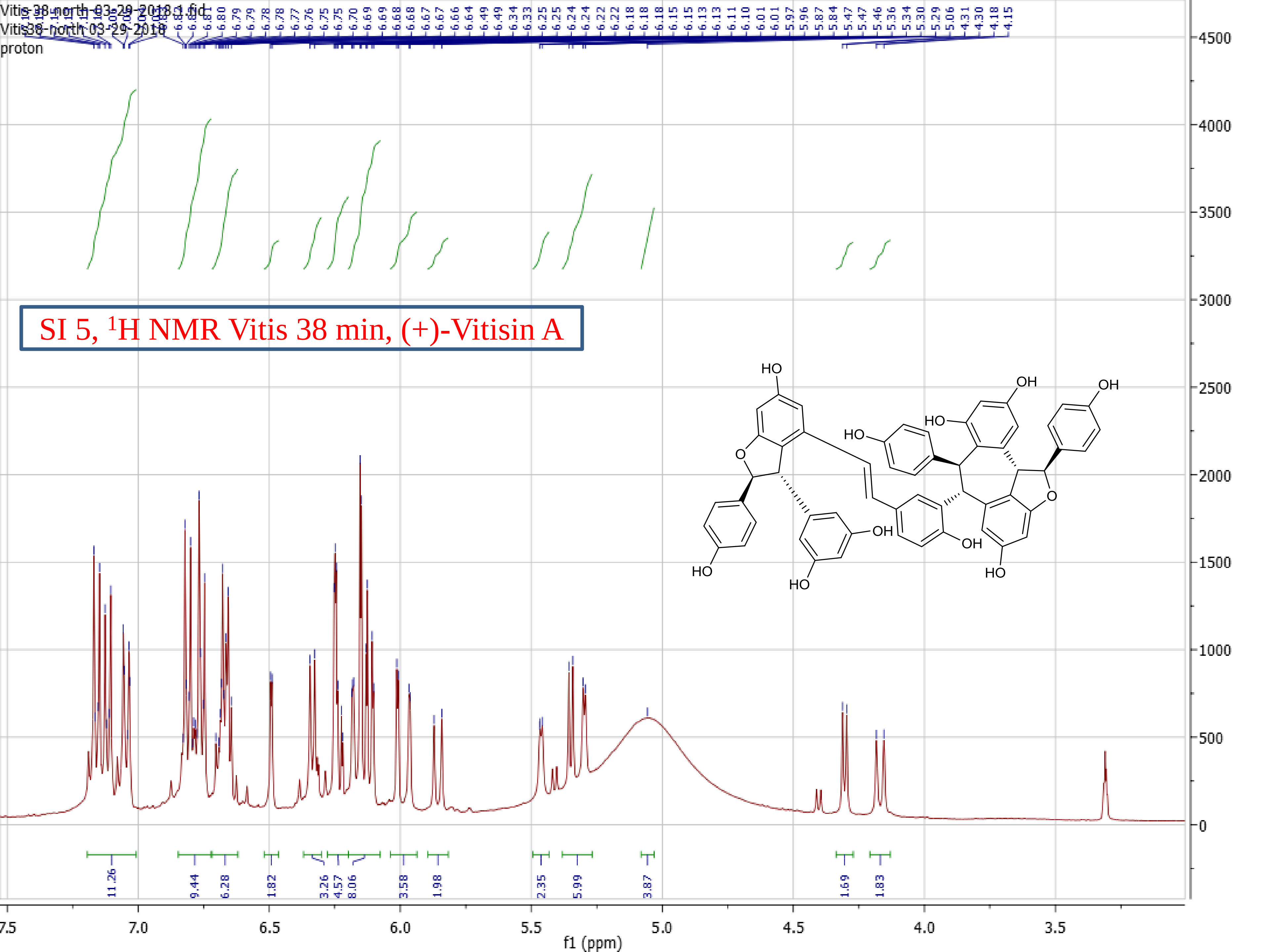

SI 5, ^{1}H NMR Vitis 38 min, (+)-Vitisin A

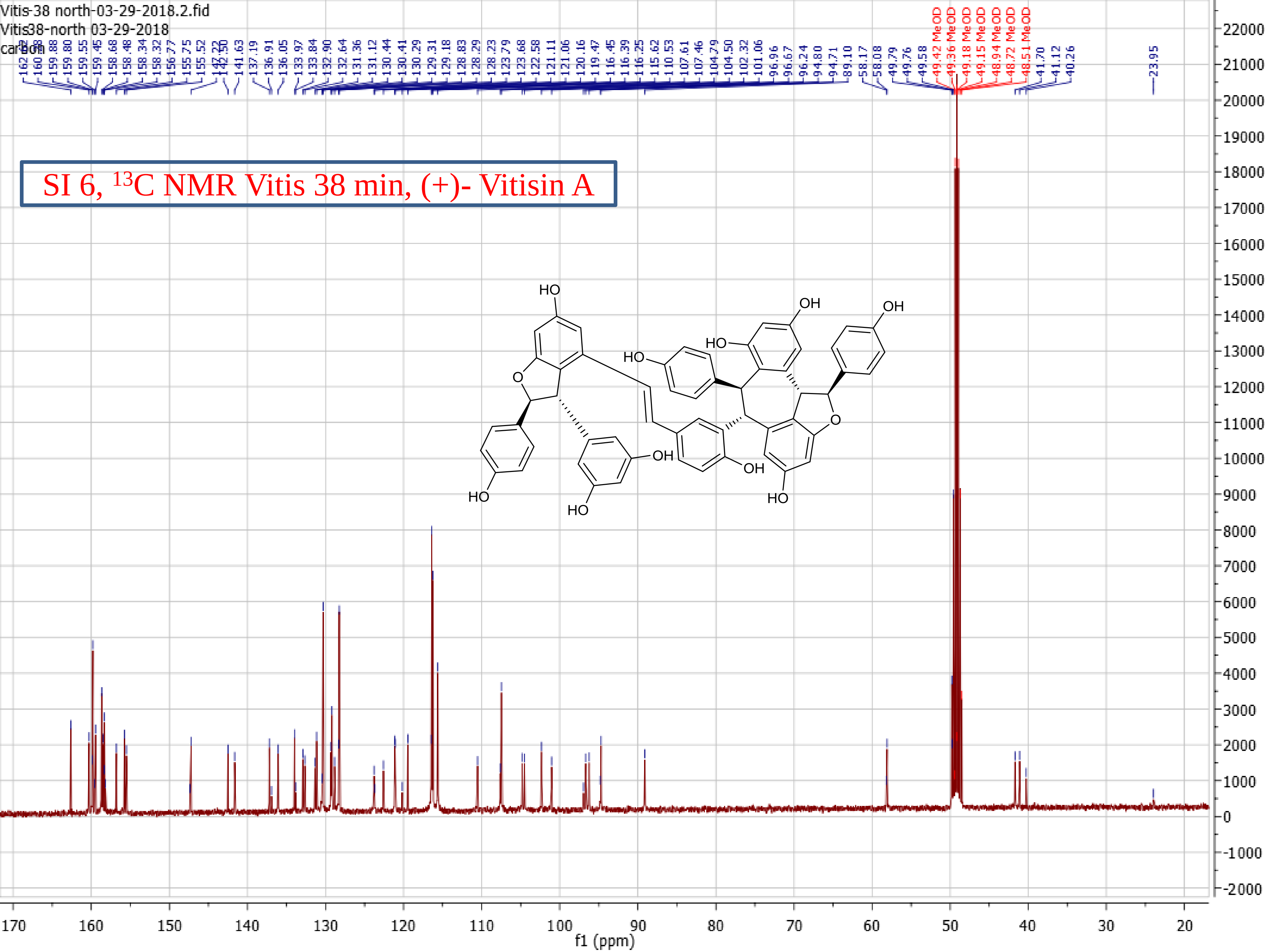

Vitis-38 north-03-29-2018.2.fid
Vitis38-north 03-29-2018
carbon
159.88
159.80
159.55
159.45
158.68
158.48
158.34
158.32
156.77
155.75
155.52
142.22
142.50
141.63
137.19
136.91
136.05
133.97
133.84
132.90
132.64
131.36
131.12
130.44
130.41
130.29
129.31
129.18
128.83
128.29
128.23
123.79
123.68
122.58
121.11
121.06
120.16
119.47
116.45
116.39
116.25
115.62
110.53
107.61
107.46
104.79
104.50
102.32
101.06
96.96
96.67
96.24
94.80
94.71
89.10
58.17
58.08
49.79
49.76
49.58
49.42 MeOD
49.36 MeOD
49.18 MeOD
49.15 MeOD
48.94 MeOD
48.72 MeOD
48.51 MeOD
41.70
41.12
40.26
23.95
SI 6, ¹³C NMR Vitis 38 min, (+)- Vitisin A
HO
OH
HO
HO
OH
O
O
OH
OH
HO
HO
HO
f1 (ppm)

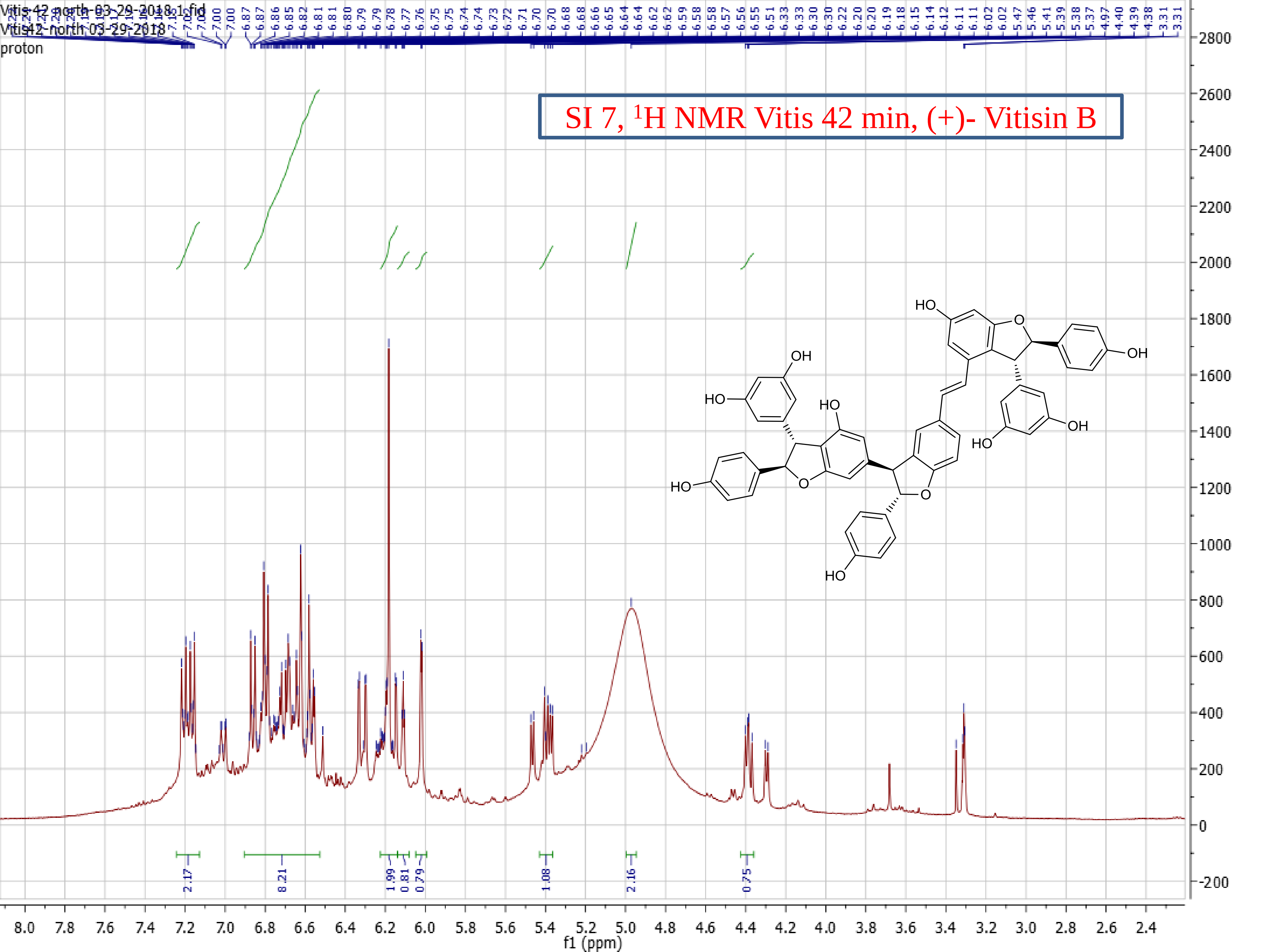
Vitis-42-north-03-29-2018.1.fid
Vitis42-north-03-29-2018
proton
7.00
7.00
6.87
6.87
6.86
6.85
6.82
6.81
6.81
6.80
6.79
6.79
6.78
6.77
6.76
6.75
6.75
6.74
6.74
6.73
6.72
6.71
6.70
6.70
6.68
6.68
6.66
6.65
6.64
6.64
6.62
6.62
6.59
6.58
6.58
6.57
6.56
6.55
6.51
6.33
6.33
6.30
6.30
6.22
6.20
6.20
6.19
6.18
6.15
6.14
6.12
6.11
6.11
6.02
6.02
5.47
5.46
5.41
5.39
5.38
5.37
4.97
4.40
4.39
4.38
3.31
3.31
SI 7, 1H NMR Vitis 42 min, (+)- Vitisin B
HO
O
OH
OH
HO
HO
OH
HO
HO
O
HO
O
HO
2.17
8.21
1.99
0.81
0.79
1.08
2.16
0.75
8.0 7.8 7.6 7.4 7.2 7.0 6.8 6.6 6.4 6.2 6.0 5.8 5.6 5.4 5.2 5.0 4.8 4.6 4.4 4.2 4.0 3.8 3.6 3.4 3.2 3.0 2.8 2.6 2.4
f1 (ppm)
2800 2600 2400 2200 2000 1800 1600 1400 1200 1000 800 600 400 200 0 -200

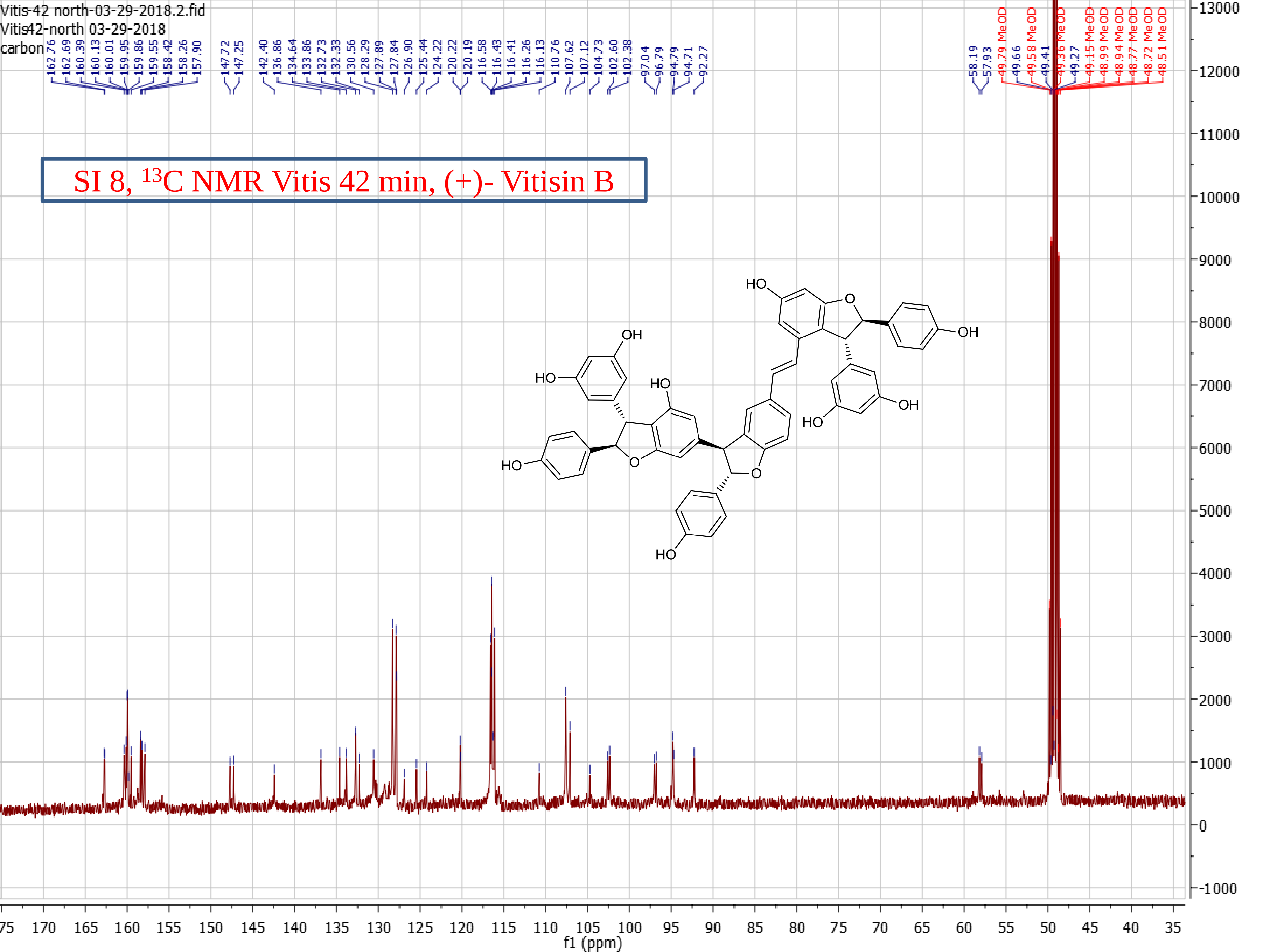

SI 8, ^{13}C NMR Vitis 42 min, (+)- Vitisin B
